# Deciphering the molecular mechanism of enhanced tumor activity of the EGFR variant T790M/L858R using melanoma cell lines

**DOI:** 10.3389/fonc.2023.1163504

**Published:** 2023-06-02

**Authors:** Hanshuang Shao, Alan Wells

**Affiliations:** ^1^ Department of Pathology, University of Pittsburgh, Pittsburgh, United States; ^2^ Pittsburgh VA Health System, Pittsburgh, PA, United States

**Keywords:** phosphorylation, invasion, migration, epidermal growth factor receptor (EGFR), resistance, TKI - tyrosine kinase inhibitor

## Abstract

**Introduction:**

The abnormal expression and mutagenesis of EGFR drives both the development and progression of a multitude of human cancers. Further mutations within the tyrosine kinase region of the EGFR subsequently contribute to resistance to targeted drugs. What is not known is how these mutations affect progression-related behaviors of cancer cells.

**Methods:**

The mutagenesis of EGFR T790M, L858R, and T790M/L858R was performed *via* oligo primer-guided polymerase chain reaction (PCR). GFP-tagged mammalian expression vectors were constructed and confirmed. Stable melanoma cell lines WM983A and WM983B expressing WT or mutant EGFRs were generated for determining the functions of WT and mutant EGFRs in migration, invasion, and resistance to doxorubicin. Immunoblotting and immunofluorescence were performed to detect the transphosphorylation and autophosphorylation of WT and mutant EGFRs and other molecules.

**Results:**

The EGFR mutant T790M/L858R showed significantly higher basal autophosphorylation in melanoma cell lines WM983A and WM983B. Overexpression of WT EGFR significantly enhanced the protein level of E-cadherin (E-cad) *via* upregulating its mRNA. In contrast, L858R significantly downregulated E-cad. Biological activity assays show that T790M/L858R presented significant enhancement *in vitro* in invasion and migration, while WT and T790M moderately inhibited invasion and migration. In WM983A cells, enhanced invasion and migration by T790M/L858R required the downstream signaling pathways through Akt and p38. T790M/L858R dramatically triggers phosphorylation of actin cross-linking protein alpha-actinin-4 in the absence of EGF. This double mutant also conferred resistance to a general chemotherapy doxorubicin through Akt but not the p38 signaling pathway.

**Conclusion:**

These findings suggest that T790M/L858R not only confers enhanced therapeutic resistance in cancer cell lines but also may promote tumor metastasis *via* its boosted downstream signaling pathways and/or direct phosphorylation of other key proteins.

## Introduction

Epidermal growth factor receptor (EGFR, also known as ErbB1), a member of the ErbB family, is a tyrosine kinase that plays crucial roles in cell proliferation, migration, differentiation, and division (1–5). EGFR consists of four major regions including highly glycosylated extracellular domain, transmembrane region, conserved cytoplasmic domain, and C-terminal tail (1, 6). The extracellular domain functions as a docking site for its ligands including epidermal growth factor (EGF) and transforming growth factor-α (TGF-α) (7). Binding of these factors to EGFR leads to homodimerization or heterodimerization with another ErbB member, followed by subsequent activation of survival and proliferation signaling pathways due to a series of transphosphorylation and autophosphorylation of tyrosines residing within its conserved cytoplasmic domain (6).

The expression of EGFR is ubiquitous but tightly regulated in healthy tissues and organs (8, 9). However, abnormal expression and dysregulated activities of EGFR drive the development of cancers in the lung, breast, prostate, colon, skin (melanoma), and others (10–15). Among all human solid cancers, the leading cause of cancer-related deaths worldwide is lung cancer (16). In the past two decades, targeted therapy based on the identification of key tumor drivers has been well developed and significantly improved the survival of lung cancer patients. Mutations in EGFR, one of the many tumor drivers, is observed in approximately 15% of non-small cell lung cancer patients. As of today, at least five generations of EGFR tyrosine kinase inhibitors (TKIs) have been developed due to acquired drug resistance of patients after a targeted therapeutic treatment for 1 to 2 years (17). For example, the first generation of EGFR-TKI targeting the inhibition of enhanced ATP binding caused by deletion of exon 19 or L858R mutation presents significant response on patients. Unfortunately, most patients became resistant to TKI after approximately 12 months because of a new mutation such as T790M. T790M impairs the binding of TKI to ATP binding dock resulting in an enhanced EGFR activity (1, 17). Thus, the third generation of TKI targeting T790M has been developed but unfortunately, patients experience drug resistance due to further mutations in the EGFR.

Although lung cancer is the leading cause of death in human cancer, the cancer with the highest EGFR mutation frequency is glioblastoma (~27%), which is approximately 12% higher than lung carcinoma (~15%) (18). The fourth highest EGFR mutation frequency in their study is skin cutaneous melanoma in which approximately 6% carry mutations in EGFR. Notably, lung carcinoma presents the highest mutation in the region of EGFR protein tyrosine kinase (712–968 aa) followed by glioblastoma and melanoma (approximately 6% for both) (18). While the mechanism of mutation-mediated resistance against target therapy was broadly investigated in lung cancer and others such as breast cancer and colon carcinoma and a great progress had been made, the other consequences of typical mutation in EGFR such as T790M, L858R, and T790M/L858R, why new resistance develops during target therapy, and how cancer cells escaped from KTI remain largely unclear.

In the present study, we chose melanoma cell line WM983A to investigate the biological function of parental and mutant EGFR, as a prelude to whether this can be targeted in those patients with EGFR mutations. Boone et al. (19) reported that EGFR appears to be involved in the progression and metastasis of a subset of melanomas using immnuohistochemistry and FISH. Among melanoma cell lines, WM983A cells isolated from the primary vertical growth tumor of a melanoma patient do not express detectable endogenous EGFR at the protein level. Thus, we use this cell line as a model to express exo-WT/mutant EGFR. We found that overexpression of WT EGFR significantly increases E-cadherin protein level *via* a transcriptional upregulation in WM983A cells. In contrast, L858R dramatically downregulates the expression of E-cadherin. Dual-mutant T790M/L858R presents enhanced invasiveness and tolerance to doxorubicin (Dox).

## Materials and methods

### Reagents and cell culture

Antibodies including EGFR, ErBB2, ErBB3, ErBB4, pEGFR(Y1173), pEGFR(Y1068), pAkt(S473), Pan-Akt, pERK, ERK, pP38, pHSP27, GAPDH, and pTyr were purchased from Cell Signaling Technology (Danvers MA). Monoclonal antibody against E-cadherin was purchased from BD Biosciences (Franklin Lakes, NJ). Monoclonal GFP antibody was purchased from Santa Cruz Biotechnology (Dallas, TX). Recombinant human epidermal growth factor (rhEGF) was purchased from Millipore-Sigma (St Louis, MO). Transfection reagent Lipofectamine 2000 was purchased from Life Technologies (Grand Island, NY). Chemical inhibitors including PD153035, LY294002, and SB203580 were purchased from R&D Systems (Minneapolis, MN). MatriGel 24-well transwells were purchased from Corning (Corning, NY). Dox was purchased from Presenius Kabi (San Diego, CA). Melanoma cell lines WM983A, WM983B, IgR3, and WM852 were cultured in a medium containing three parts of DMEM (1 g L^−1^ glucose) and one part of L15 in addition to 10% fetal bovine serum and 1× pen/strip antibiotics.

### Mutagenesis

The mutagenesis of EGFR T790M, L858R, and T790M/L858R was performed *via* oligo primer-guided polymerase chain reaction (PCR) and confirmed by Sanger DNA sequencing. GFP-tagged mammalian expression vectors of WT and mutant EGFRs were constructed and identified by restriction enzyme analysis.

### Transfection and selection of stable cell lines

Melanoma WM983A cells were plated in a six-well plate and cultured in complete growth medium overnight to get approximately 60% cell confluence at transfection. The next day, 10 µl of Lipofectamine 2000 and 4 µg of plasmid DNA were diluted in 250 µl of Opti-medium with low serum, respectively, and then gently mixed. After incubating at room temperature for 30 min, the mixture was slowly added into a cell culture well containing fresh quiescence media with 0.5% dialyzed fetal bovine serum for an additional 16-h incubation at 37°C in a humidified incubator with 5% CO_2_. For the selection of stable colonies, cells were incubated in the Opti-medium containing the complex of Lipofectamine 2000 and plasmid DNA for 4 h and then switched to normal complete growth media. After 48 h of culture, cells were split at a ratio of 1 to 50 into selection medium containing 1,500 µg/ml G418 to allow the formation of monoclonal colonies.

### Immunoblotting

Cells were lysed in in RIPA buffer in the presence of 1× protease inhibitors cocktails set V (Temecula CA) followed by incubating on ice for 5 min prior to sonicating briefly. Samples were transferred to 1.7-ml microtubes for centrifugation at 13,000*g* at 4°C for 30 min. The supernatant of each was carefully transfected to a new tube, and the concentration of total soluble proteins was determined using BCA™ Protein Assay (Thermo Scientific™ Pierce™, Rockland, IL). For each sample, 10 µg of total proteins was mixed with one-fifth volumes of 5× sodium dodecyl sulfate (SDS) sample buffer with β-mercaptoethanol and boiled for 5 min prior to loading on appropriate concentration of acrylamide gel based on the size of interest protein. After gel running, proteins in SDS gel were transferred to polyvinylidene difluoride (PVDF) membrane followed by incubating the membrane with 5% fat-free milk at room temperature for half hour to block non-specific binding. Then, membrane was incubated with indicated primary antibodies on a shaker at a slow speed in cold room overnight. Next day, the membrane was thoroughly washed with 1× Tris buffered saline (TBS) containing 0.1% Tween-20 and then incubated with diluted secondary antibody at room temperature for 45 min followed by complete washing. Finally, the target protein was developed using ECL reagents and a processing machine.

### Immunofluorescence

Cells were fixed with 3.7% formaldehyde in phosphate buffered saline (PBS) at room temperature for 30 min. After washing to remove residual formaldehyde, cells were permeabilized with 0.2% Triton X-100 in PBS on ice for 5 min followed by complete wash. Then, cells were incubated with 1% bovine serum albumin in PBS at room temperature for 30 min followed by 1-h incubation with primary antibodies at appropriate dilution. Cells were thoroughly washed and then incubated with conjugated secondary antibody diluted at a ratio of 1 to 2,000 at room temperature for 1 h followed by complete wash. After a brief incubation in diluted DAPI and complete wash, the coverslip was mounted on the slide for imaging under a fluorescent confocal microscope at the Center of Biological Images of the University of Pittsburgh.

### *In vitro* scratch wound assay

Cells were plated and allowed to grow until confluence. Then, the monolayer of cells was carefully and slowly scratched around the center of well using a wood scraper to create a wound area at approximately 3 mm in width. Floating cells were carefully and completely washed away with Ca^++^/Mg^++^ PBS. To make sure images were taken at time 0 h and 24 h at the same positions, two black vertical lines with a scratched edge were marked at the bottom of each well using a marker pen. Then, images involving each mark line were taken as time 0 h. After 24-h incubation, images at the same positions were retaken. Finally, the relative size of each wound was measured using the ImageJ software at time 0 h and 24 h, respectively. After dividing the difference of area size by the height of the wound area, the value means the net relative migratory units.

### MatriGel transwell invasion assay

MatriGel transwells, stored at −20°C, were thawed and warmed to room temperature followed by adding 500 µl of quiescence medium containing 0.5% dialyzed fetal bovine serum to the transwell and bottom chamber, respectively. After equilibrating at 37°C for 2 h in a humidified incubator with 5% CO_2_, 50,000 cells suspended in 500 µl of quiescence medium were applied to the transwell and 750 µl of complete growth medium was added to the bottom chamber for an additional 24 h or 48 h of incubation. The medium in the transwell chamber was carefully aspirated and all cells and MatriGel on the top surface of the transwell membrane were thoroughly removed using a loose cotton swab. Then, cells invaded through the MatriGel and membrane and attached to the bottom surface of the transwell membrane were fixed by 3.7% formaldehyde in PBS. For the stain of nuclei by DAPI, fixed cells were briefly permeabilized using Triton X-100 and stained with DAPI. Finally, invaded cells were randomly imaged and all cells on the entire membrane were counted under a microscope.

### Cell death assay

The cytotoxicity of Dox for cells was determined by cell death assay. Briefly, cells were cultured in complete growth medium containing an appropriate concentration of Dox. After 24 h or 48 h of incubation, the medium containing Dox and floating dead cells were removed. Attached cells were carefully washed prior to being fixed with 3.7% formaldehyde at room temperature for 30 min. Cells were then washed and stained with 0.5% crystal violet followed by washing, imaging, and then extracting by 2% SDS solution. The optical density of extraction at 560 nm wavelength was determined using a spectrophotometer.

## Results

### EGFR mutants present enhanced basal autophosphorylation

**Figure 1:**
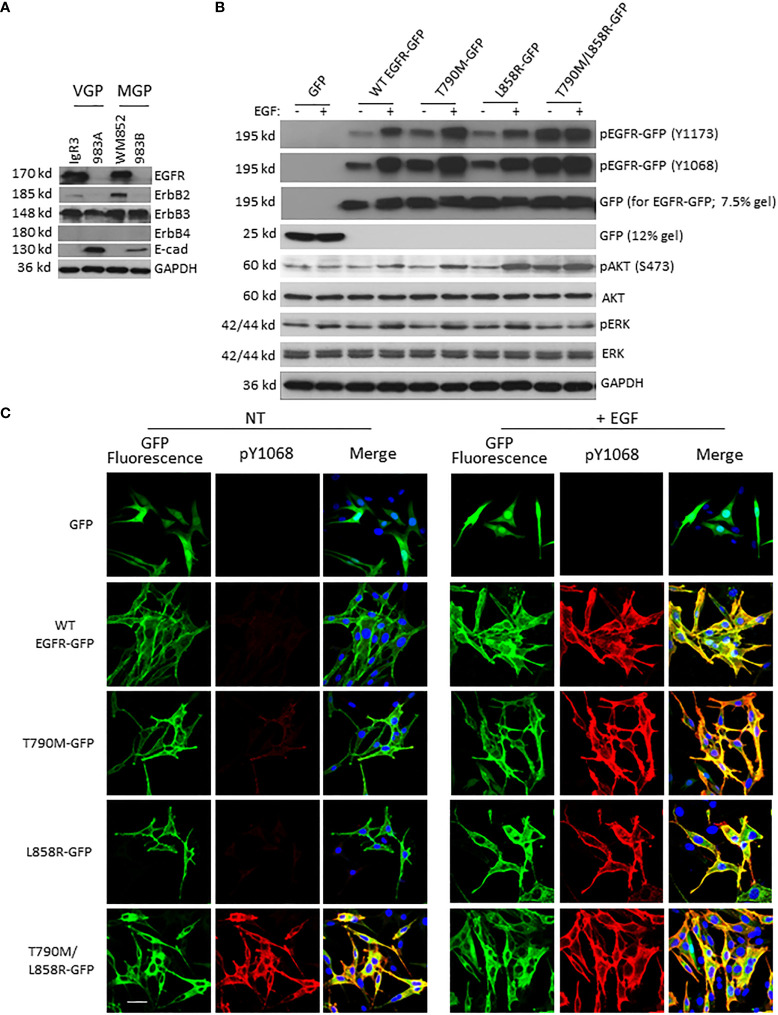
EGFR mutants present enhanced autophosphorylation in WM983A cells. **(A)** Immunoblottings of indicated proteins from melanoma cell lines. **(B)** Immunoblottings of indicated proteins from WM983A cells stably transfected with GFP or GFP-tagged WT/mutant EGFR and treated with 10 nM EGF for 15 min prior to harvesting cells. In **(A, B)**, representative results of three independent experiments are shown. **(C)** Representative immunofluorescent images of stable WM983A cells stimulated with EGF and immunostained with phospho-EGFR(Y1068) antibody (red) and DAPI (blue). Scale bar = 20 µm. Shown are representatives from three independent experiments.

**Figure 2:**
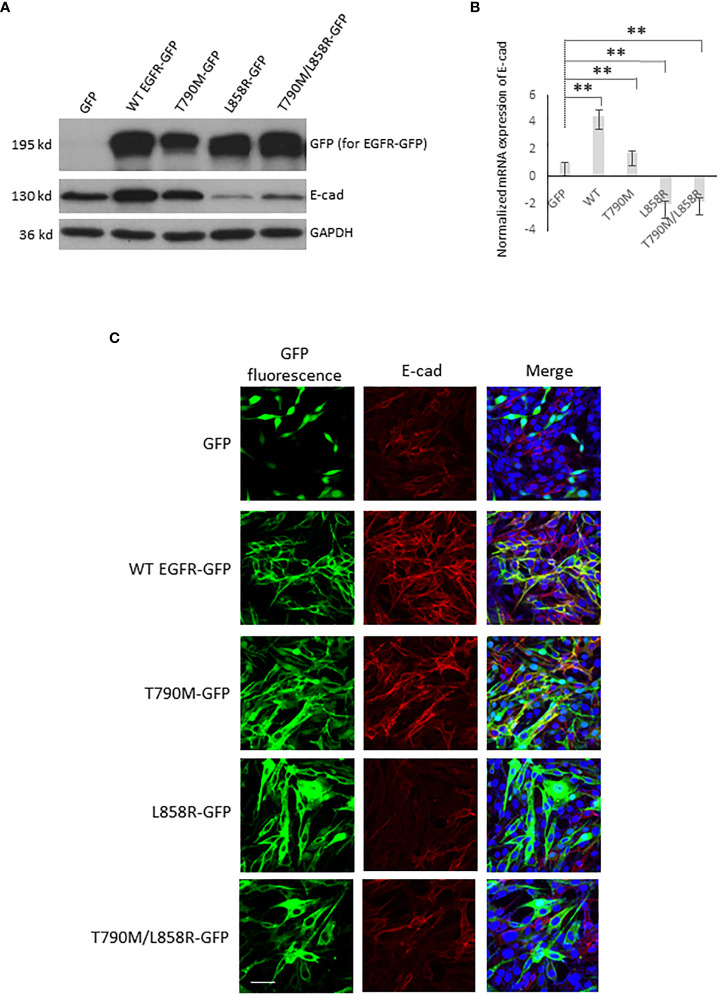
EGFR alters the E-cadherin level in WM983A cells. **(A)** Immunoblottings of indicated proteins from stable WM983A cells expressing GFP or GFP-tagged WT/mutant EGFR. Shown are representative results of three independent experiments. **(B)** Normalized mRNA expression of E-cad measured by quantitative PCR. GAPDH is used for normalization. Data are mean ± SD of three independent experiments. Statistical analysis was performed using Student’s *t*-test. **p < 0.01. **(C)** Representative immunofluorescent images of stable WM93A cells immunostained with E-cad antibody (red) and DAPI (blue). Scale bar = 20 µm. Shown are representatives from three independent experiments.

To study the function of wild type (WT) and mutant EGFRs, we chose melanoma cell lines WM983A in which their endogenous EGFR protein levels are almost non-detectable compared to other melanoma lines IgR3 and WM852 (**Figure 1A**). The effect of endogenous EGFR would be very limited if any, as we did not detect any phosphorylated EGFR when EGF stimulation was applied (**Figure 2A**, the first two lanes). WM983A is from a melanoma in radial growth phase, and thus can be promoted to invasiveness and dissemination. After transfection and selection of WM983A cells, stable cell lines expressed WT and mutant EGFRs. Immunoblotting showed that all WT and mutants were expressed correctly based on their molecular weight (**Figure 1B**). Consistent with literature reports, T790M and L858R presented enhanced EGFR phosphorylation at both tyrosine 1173 and 1068 (**Figure 1B**) compared to WT in the absence of EGF treatment. Surprisingly, T790M/L858R presented a much higher level of autophosphorylation. Whereas EGF exposure significantly enhanced the phosphorylation of WT, T790M, and L858R, T790M/L858R was not further tyrosyl-phosphorylated, suggesting that most of the receptors are phosphorylated in the absence of exogenous ligand. As a result, the phosphorylation level of Akt in T790M/L858R-expressing cells were significantly increased in the absence of EGF treatment while T790M and L858R are only slightly changed compared to GFP-expressing cells.

While tyrosyl-phosphorylation is a marker of EGFR activation, the downstream pathways need to be verified for activation status. It appears that the level of phosphorylation of Akt or ERK in cells expressing the EGFR with individual mutation was not increased over that of cells with GFP or WT in the absence of EGF stimulation (**Figure 1B**). EGF exposure increased pAkt and pERK levels in cells expressing WT, T790M, or L858R. Interestingly, cells expressing the T790M/L858R double mutant had elevated levels of pAkt but not pERK; in both cases, exposure to EGF did not increase the level of activation, suggesting that the double mutant was signaling downstream independently of EGF.

To further confirm the enhanced autophosphorylation of EGFR mutants, cells were immunostained with EGFR phospho-Y1068 antibody. As shown in **Figure 1C**, in line with immunoblotting result, WT did not show apparent yellow co-immunofluorescence stain in the absence of EGF treatment. In contrast, T790M and, to a lesser extent, L858R presented some detectable basal phosphorylation. The T790M/L858R double mutant was strongly phosphorylated in the absence of EGF. EGF led to high-level phosphorylation in cells expressing the WT and single-mutant EGFR. As expected, the autophosphorylated EGFR (WT or mutant) was found to be predominantly internalized.

Our previous findings revealed that EGFR directly phosphorylates alpha-actinin-4 (ACTN4) at tyrosines (20). Thus, we tested if the phosphorylation of ACTN4 in T790M/L858R-expressing WM983A cells is higher than that in WT-, T790M-, or L858R-expressing cells. Indeed, as shown in **Figure S1**, T790M/L858R triggered the strongest phosphorylation of ACTN4 in the absence of EGF compared to others.

To determine whether the functions of WT and mutant EGFR are not cell line-dependent, we transiently transfected WM983B cells with these plasmids. Immunoblotting (**Figure S2A**) and immunofluorescence (**Figure S2B**) analyses of transiently transfected WM983B cells show that WT and mutant EGFRs in WM983B function similarly to that in WM983A cells. Unfortunately, we failed to establish a stable WM983B cell line expressing T790M/L858R, for uncertain reasons.

Taken together, these results suggest that WT and mutant EGFRs work functionally in WM983A and WM983B cells and T790M/L858R presents significant enhancement of autophosphorylation independent of ligand.

### WT and mutant EGFR alter E-cadherin level in WM983A and WM983B cells

Critical to cancer cell migration and invasion is the downregulation of E-cadherin (16, 21–24). EGFR has been suggested to directly interact with E-cadherin (E-cad) in many cells (25, 26). We next tested whether overexpression of WT and mutant EGFRs affect the protein level of E-cad in stable-expressing WM983A and WM983B cells. Interestingly, while T790M just moderately lifted the protein level of E-cad, the upregulation of E-cad in WT is multi-fold in both WM983A (**Figure 2A**) and WM983B cells (**Figure S3A**). Surprisingly, L858R dramatically downregulated the protein level of E-cad. T790M/L858R also decreased E-cad even though at a less content compared to L858R in WM983A cells.

Activated EGFR can lead to reduced E-cad protein levels (27–29). However, we needed to determine whether EGFR-mediated changes to E-cad could be through transcriptional regulation (26). Thus, we determined the mRNA level of E-cad using quantitative PCR. As shown in **Figure 2B**, in WM983A cells, the mRNA level of E-cad in both WT and T790M cells were higher than GFP-expressing cells. In contrast, both L858R and T790M/L858R downregulated the transcription of E-cad mRNA.

To further confirm the changes to E-cad caused by overexpression of EGFR in WM983A and WM983B cells, the immunofluorescence of E-cad localization was performed. **Figures 2C**, **S3B** show that WT- and T790M-expressing cell lines presented strong E-cad fluorescence (red) and co-immunofluorescence of E-cad (red) and EGFR-GFP (green) in yellow. Notably, E-cad mainly localizes at cell–cell junctions as would be expected for functional E-cad. The fluorescence of E-cad in L858R-expressing cells was significantly lower compared to GFP-expressing cells. As a result of the increase of E-cad level in WT-expressing WM983A cells, we observed cell clusters during routine cell culture while L858R-expressing cells with significantly downregulated E-cad were individually sparse (**Figure S4**). Taken together, these results suggest that overexpression of WT or mutant EGFRs affects the level and functionality of E-cad *via* transcriptional regulation.

### T790M/L858R enhances migration and invasion of WM983A cells

**Figure 3:**
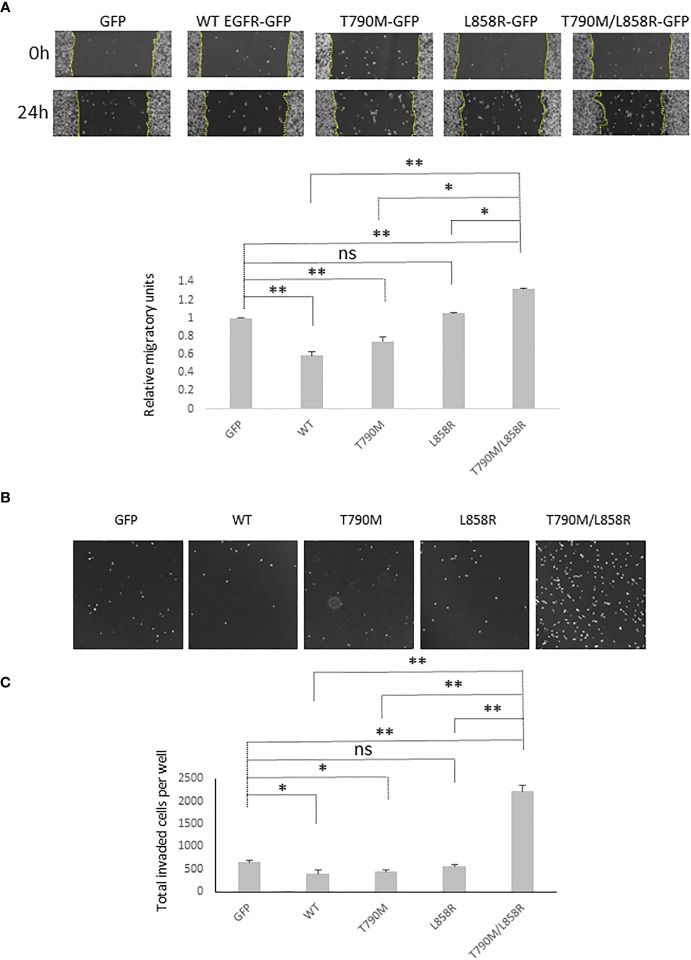
EGFR promotes the migration and invasiveness of WM983A cells. **(A)** Representative images of the whole wound area of confluent WM983A cells stably expressing GFP or GFP-tagged WT/mutant EGFRs taken at time 0 h and 24 h. Migration speed has been quantified and then normalized to GFP-expressing cells. Data are mean ± SD of three independent experiments. **(B)** Representative images of nuclei of invaded cells on the bottom surface of transwell membrane. **(C)** Quantitative results of invaded cells after 48-h incubation per entire transwell membrane. Data are mean ± SD of three independent experiments. In **(A, C)**, statistical analysis was performed using Student’s *t*-test. *p < 0.05, **p < 0.01, ns, not significant.

As downstream signaling from EGFR and E-cad functionality play vital roles in the migration and invasion of cells, we next tested how overexpression of WT or mutant EGFRs affected the migration and invasion of WM983A cells. Overexpression of WT significantly inhibited the migration of WM983A cells compared to GFP-expressing cells (**Figure 3A**). T790M also inhibits the migration of WM983A cells but not as much as WT. In contrast, T790M/L858R significantly promotes the migration of WM983A cells.

MatriGel transwell invasion assays showed that WT and T790M significantly impede the invasion of WM983A cells through the MatriGel (**Figures 3B, C**). We observed a huge enhancement on the invasion of T790M/L858R-expressing cells. These results suggest that WT and T790M slow down but T790M/L858R promotes the migration and invasion of WM983A cells, in line with expectations from changes in E-cad functioning.

### T790M/L858R enhances the resistance of melanoma cells to doxorubicin treatment

**Figure 4:**
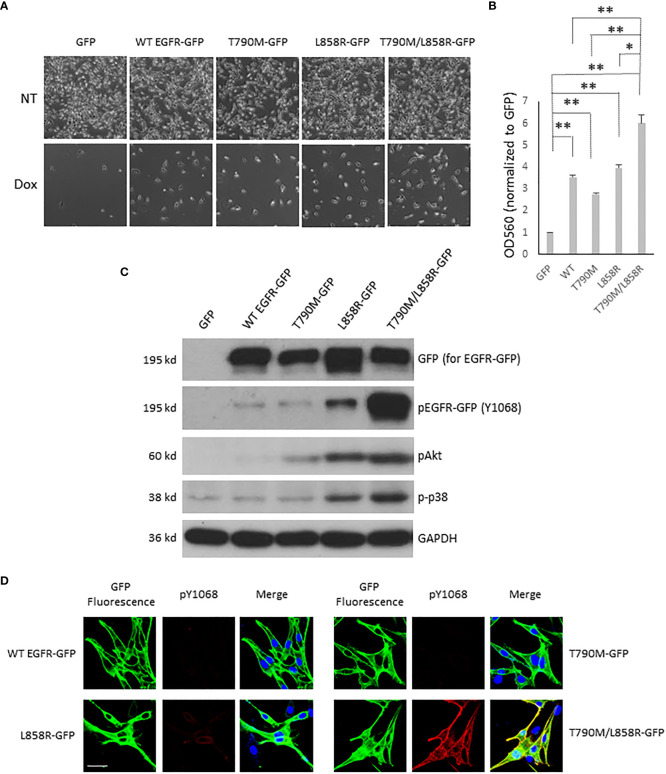
EGFR enhances the resistance of WM983A cells to doxorubicin challenge. **(A)** Representative images of WM983A cells stably expressing GFP or GFP-tagged WT or mutant EGFR. Top panel: no Dox; Bottom panel: attached cells after treatment with 12 µM of Dox for 48 h. **(B)** Quantitative results of cells in **(A)** using crystal violent stain method. Data are mean ± SD of three independent experiments. Statistical analysis was performed using Student’s *t*-test. *p < 0.05, **p < 0.01, ns: not significant. **(C)** Immunoblottings of indicated proteins from stable WM983A cells. Shown are representatives from three independent experiments. **(D)** Representative images of stable WM983A cells immunostained with phospho-EGFR (Y1068) antibody (red) and DAPI (blue). Scale bar = 20 µm. Shown are representatives from three independent experiments.

Chemotherapeutic resistance often occurs after EGFR mutagenesis-related cancer patients take targeted-therapy medication for a median time of 12 months (17, 30, 31). The main reason in most cancer patients is a second and even a third mutational event. To determine the chemo-tolerance of WM983A and WM983B cells in which WT or mutant EGFR is either stably or transiently expressed, we first determined the chemo-tolerance of parental WM983A and WM983B cells in different concentrations of Dox. WM983A cells were more resistant to Dox than WM983B cells. Most WM983B cells did not survive in the treatment of 3 µM of Dox but a large part of WM983A cells remained attached to the culture dish without a significant change in cellular morphology (**Figure S5**). Next, we treated WM983A cells expressing WT or mutant EGFR with 12 µM of Dox for 48 h prior to imaging or fixing by formaldehyde followed by crystal violet stain and extraction. As shown in **Figures 4A, B**, all WT and mutant EGFR-expressing cells were more resistant to Dox than cells expressing GFP. Notably, T790M/L858R presented the strongest resistance compared to WT, T790M, and L858R.

The high resistance of T790M/L858R cells could be due to the strong phosphorylation of T790M/L858R at tyrosines in normal growth medium (**Figures 4C, D**), resulting in activation of the survival-associated Akt and p38. To further confirm the function of WT and mutant EGFR concerning the increased resistance to Dox, indicated concentrations of Dox were added to WM983B cells that transiently overexpressed EGFR for a 24-h incubation period prior to imaging. As shown in **Figure S6A**, most of the GFP-expressing cells died in the 3 µM Dox treatment and no cells survived at a concentration of 4 µM. As we expected, some WT or mutant EGFR-expressing cells were able to tolerate the higher concentration of Dox. Among mutants, some T790M/L858R-expressing cells survived in the face of 5 µM Dox. To quantify the Dox tolerance of WT and mutants expressing WM983 cells, immunoblotting of GFP was performed on survival cells after Dox treatment. As shown in **Figures S6B, C**, we observed more T790M and L858R in the lanes of Dox treatment than WT. The density of L858R band after Dox treatment was higher than T790M. Cells expressing T790M/L858R presented a light band but its normalized percentage was much higher than WT, T790M, and L858R in both 3 and 4 µM concentrations of Dox. Taken together, these results suggest that T790M/L858R confers enhanced resistance to WM983A and WM983B cells in Dox treatment.

### T790M/L858R promotes migration and invasion *via* Akt and p38 signaling pathways

**Figure 5:**
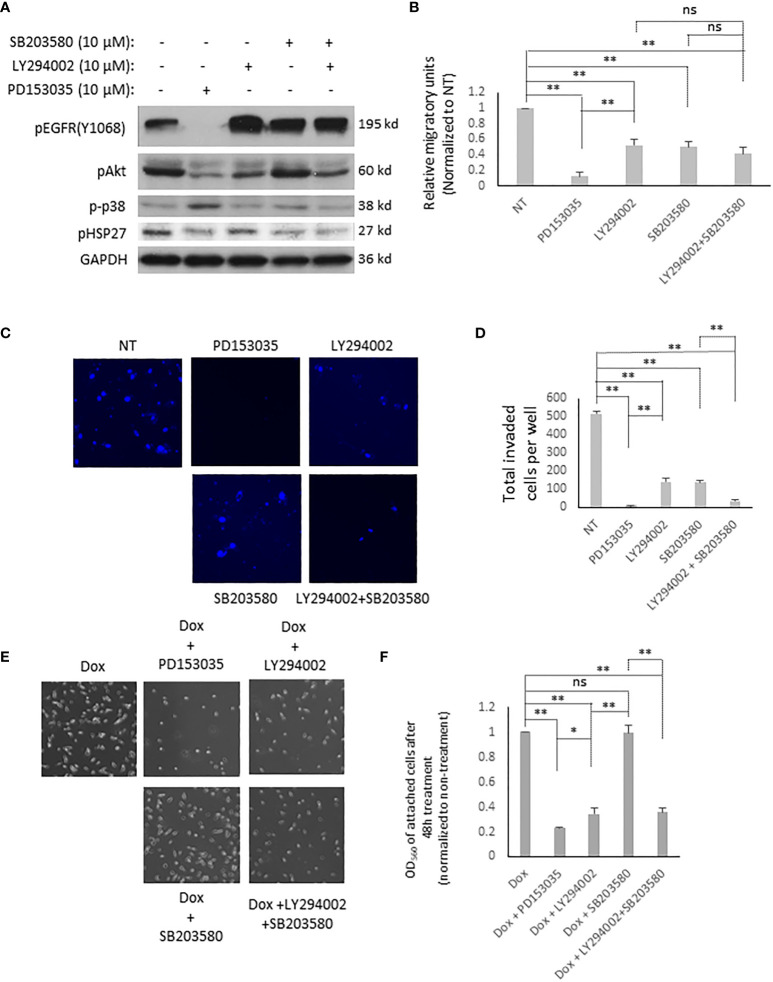
EGFR T790M/L858R activates different signaling pathways for its functions in WM983A cells. **(A)** Immunoblottings of indicated proteins from stable WM983A cells. Shown are representative results of three independent experiments. **(B)** Quantitative results of stable WM983A cells expressing T790M/L858R in the presence of indicated inhibitors. **(C)** Representative images of invaded stable T790M/L858 WM983A cells. **(D)** Quantitative results of **(C)**. Numbers stand for all invaded cells after 24 h incubation per entire transwell membrane. **(E)** Representative images of stable T790M/L858 WM983A cells treated with 12 µM of Dox and 10 µM of indicated inhibitors for 48 h. **(F)** Quantitative results of **(E)**. Data are mean ± SD of three independent experiments. Statistical analysis was performed using Student’s *t*-test. *p < 0.05, **p < 0.01, ns, not significant.

Activated EGFR triggers a plethora of downstream signaling pathways (32). As the above data showed that T790M/L858R significantly enhanced the migration, invasion, and resistance to Dox treatment in WM983A cells, we next determined which downstream signaling pathways might be involved. **Figure 4C** shows that cells expressing T790M/L858R presented significantly elevated phosphorylation at tyrosine 1068, and Akt at serine 473 and p38. Thus, we treated T790M/L858R-expressing WM983A cells with PD153035, LY294002, and SB203580, which are specific inhibitors for EGFR, Akt, and p38, respectively. Immunoblottings showed that the phosphorylation of EGFR at tyrosine 1068 was completely blocked by PD153035 and LY294002 significantly reduced the phosphorylation of Akt at serine 473 (**Figure 5A**). Notably, SB203580, a specific p38 inhibitor, did not directly inhibit the phosphorylation of p38 but did decrease phosphorylation of HSP27, a downstream target of p38.

As shown in **Figures 5B–F**, EGFR inhibition using PD153035 significantly inhibited the migration, invasion, and the resistance to Dox of T790M/L858R-expressing WM983A cells. LY294002 also presents significant inhibitory effects but not to the same extent as PD153035. Surprisingly, SB203580 significantly blocked the migration and invasion of T790M/L858R-expressing WM983A cells but did not decrease the resistance to Dox, suggesting that the p38 signaling pathway is not required for the enhanced resistance to Dox (**Figures 5E, F**). Furthermore, the combination of LY294002 and SB203580 presents a partial enhancement in the inhibition of cell migration compared to LY294002 or SB203580 mono treatment but still not as efficiently as PD153035 (**Figure 5B**). Interestingly, the effect on the inhibition of the invasion was significantly boosted when LY294002 and SB203580 were simultaneously applied to cells (**Figure 5C**). PD153035 almost completely blocked the migration and invasion, but not the resistance to Dox, indicating that other signaling pathway(s) or molecules could be involved in its enhanced resistance. Taken together, these results suggest that T790M/L858R promotes the migration of WM983A cells *via* the Akt and p38 pathways, the invasion completely *via* the Akt and p38 pathways, and the resistance to Dox only *via* the Akt pathway.

## Discussion

Melanoma cell lines WM983A and WM983B were isolated from the vertical growth phase and metastatic liver of the same one patient, respectively. While other melanoma cell lines express variable levels of EGFR (23), the EGFR protein in both WM983A and WM983B cells is almost not detectable compared to other cell lines (**Figure 1A**). During the selection of the stable cell line for T790M/L858R, we unfortunately failed to establish stably expressing WM983B cells. This was probably due to the significantly enhanced autophosphorylation of T790M/L858R at tyrosines such as Y1068 and Y1173. On one hand, the highly phosphorylated T790M/L858R becomes sensitive to degradation, resulting in a gradual loss of T790M/L858R-GFP protein during selection; on the other hand, autophosphorylation of T790M/L858R triggers a strong activation of downstream signaling such as Akt, p38, and probably others that limit cell growth or cause a cell death in WM983B cells. Indeed, we observed that stable WM983A cells gradually lose T790M/L858R-GFP during routine cell culture (data not shown). Besides melanoma cells, we also tried to express this double mutant in the poorly metastatic breast cancer cell line MCF-7. We also failed to establish a stable MCF-7 cell line for T790M/L858R. Furthermore, it was very difficult for us to sort positive polyclonal colonies due to the high level of E-cad-mediated cell–cell clusters in MCF-7 culture. It should be noted that we could establish stable cell lines for both WM983B and MCF7 with the WT EGFR and the singular mutants. However, this limitation of the double mutant EGFR is the focus of ongoing experiments to determine the precise signaling cascade that is incompatible with stable expression.

The direct interaction between EGFR and E-cad has been reported (33, 34). In the present study, we found that overexpression of WT or T790M in WM983A cells increased the expression of E-cad *via* a transcriptional regulation. Surprisingly, L858R and T790M/L858R downregulated E-cad. This finding suggests that T790M and L858R play opposite roles in the regulation of E-cad expression at the transcriptional level. L858R limits the role of T790M in the upregulation of E-cad in dual-mutant T790M/L858R. As a result, the migration and invasiveness of stable WT and T790M WM983A cells migrated much slower than GFP-expressing cells although T790M presents enhanced autophosphorylation. Surprisingly, L868R significantly downregulated the E-cad in WM983A cells but did not increase their migration and invasiveness, suggesting that an appropriate level of E-cad is required for autophosphorylated EGFR-mediated enhanced migration and invasiveness of WM983A cells. However, dual-mutant T790M/L858R played a significant positive role in the migration, invasion, and resistance to Dox. Our data strongly suggest that this is due to its enhanced downstream signaling pathways. Dual inhibition of the p38 and Akt pathways phenocopied inhibition of EGFR in blocking invasiveness but not fully in blocking migration. As shown in **Figure S1**, we detected very strong phosphorylation of ACTN4 in stable T790M/L858R cells. ACTN4 has been previously shown to be phosphorylated when cells are stimulated by EGF (35). This implies that there might be other unknown proteins that can also be highly phosphorylated directly or indirectly *via* enhanced autophosphorylation of T790M/L858R. This definition of an additional pathway for migration lies beyond the scope of the present study.

The quenching of EGFR activation by PD153035 (**Figure 5A**) did not completely abolish the resistance of T790M/L858R cells to Dox, suggesting that there might be other molecules or signaling pathway(s) involved in its acquired drug resistance (17). This is probably why the second generation of KTIs that only target the tyrosine kinase activity of T790M/L858R are not able to kill all T790M/L858R-positive resistant cells generated in parents who carry the L858R mutant and are under target therapy. Furthermore, once cancer cells carrying the double mutation T790M/L858R escape from target therapy, they might have increased metastatic ability to other distal organ(s) due to the enhanced migratory and invasive capabilities. The effect of both L858R and T790M/L858R on the downregulation of E-cad in WM983A cells resulting in a more dispersed behavior implies that there might be an epithelial–mesenchymal transient before and after target therapy. Our findings here provide new impetus to develop new generations of target therapy for mutant EGFR-related human cancers in the future.

## Data availability statement

The original contributions presented in the study are included in the article/**Supplementary Material**. Further inquiries can be directed to the corresponding author.

## Author contributions

HS conceived of the experiments, performed the experiments, analyzed the data and wrote the manuscript. AW helped in the concept, analyzed the data, edited the manuscript and provided for the funding. All authors contributed to the article and approved the submitted version.

## Supplementary material

Supplementary Figure 1 Immunoblotting of ACTN4-GFP and EGFR with monoclonal pTyr antibody (top panel). ACTN4-GFP transiently co-expressed with GFP tag free WT or mutant EGFR in WM983A cells was immunoprecipitated using GFP antibody. Top bands stand for pTyr of EGFR co-immunoprecipitated with ACTN4-GFP. Bottom bands stand for pACTN4-GFP at tyrosines. The Bottom panel represents transferred ACTN4-GFP protein on PVDF membrane. The membrane was stained using Coomassie G250 and destained after immunoblotting to show ACTN-GFP protein. Shown are representative results of three independent experiments.

Supplementary Figure 2 EGFR mutants present enhanced autophosphorylation in WM983B cells. **(A)** Immunoblottings of indicated proteins from WM983B cells transiently transfected with GFP or GFP-tagged WT/mutant EGFR and treated with 10nM EGF for 15 min prior to harvesting cells. Shown are representative results of three independent experiments. **(B)** Representative immunofluorescent images of transiently transfected WM983B cells stimulated with EGF and immunostained with phospho-EGFR(Y1068) antibody (Red) and DAPI (blue). Scale bar=20 µm. Shown are representatives from three independent experiments.

Supplementary Figure 3 EGFR alters the E-cadherin level in WM983B cells. **(A)** Immunoblottings of indicated proteins from stable WM983B cells expressing GFP or GFP-tagged WT/mutant EGFR. Shown are representative results of three independent experiments. **(B)** Representative immunofluorescent images of stable WM93B cells immunostained with E-cad antibody (Red) and DAPI (blue). Scale bar=20 µm. Shown are representatives from three independent experiments.

Supplementary Figure 4 Representative images of stable WM983A cells expressing GFP or GFP-tagged EGFR during routine cell cultures. Images were randomly taken under optical microscope at 10x magnification.

Supplementary Figure 5 Representative images of WM983A and WM983B cells with or without Dox treatment at indicated concentrations for 48h. Images were randomly taken under optical microscope at 10x magnification.

Supplementary Figure 6 EGFR enhances the resistance of WM983B cells to doxorubicin challenge. **(A)** Representative images of WM983B cells transiently expressing GFP or GFP-tagged WT or mutant EGFR treated with indicated concentrations of Dox for 48h. **(B)** Immunoblottings of GFP-tagged EGFR proteins from transiently transfected WM983B cells treated with Dox at 3µM or 4µM for 48h. Shown are representatives from three independent experiments. **(C)** Quantitative results of **(B)**. Data are mean of ± SD of three independent experiments.
