# Supplementary figures and images for: Deciphering the molecular mechanism of enhanced tumor activity of the EGFR variant T790M/L858R using melanoma cell lines
Source: Front Oncol. 2023 Jun 2;13:1163504. doi: 10.3389/fonc.2023.1163504 (PMC10272518; doi:10.3389/fonc.2023.1163504)

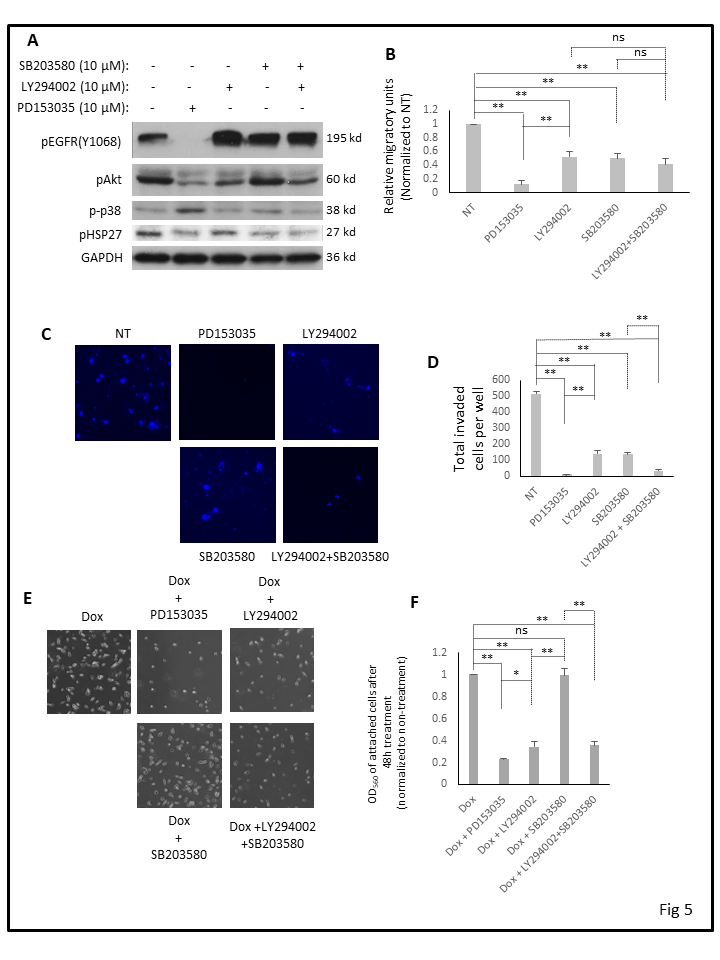

Supplement: Supplementary Figure 1 — Immunoblotting of ACTN4-GFP and EGFR with monoclonal pTyr antibody (top panel). ACTN4-GFP transiently co-expressed with GFP tag free WT or mutant EGFR in WM983A cells was immunoprecipitated using GFP antibody. Top bands stand for pTyr of EGFR co-immunoprecipitated with ACTN4-GFP. Bottom bands stand for pACTN4-GFP at tyrosines. The Bottom panel represents transferred ACTN4-GFP protein on PVDF membrane. The membrane was stained using Coomassie G250 and destained after immunoblotting to show ACTN-GFP protein. Shown are representative results of three independent experiments. [file Image_1.tif]

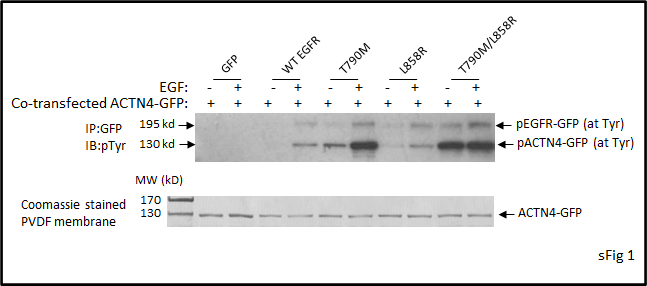

Supplement: Supplementary Figure 2 — EGFR mutants present enhanced autophosphorylation in WM983B cells. (A) Immunoblottings of indicated proteins from WM983B cells transiently transfected with GFP or GFP-tagged WT/mutant EGFR and treated with 10nM EGF for 15 min prior to harvesting cells. Shown are representative results of three independent experiments. (B) Representative immunofluorescent images of transiently transfected WM983B cells stimulated with EGF and immunostained with phospho-EGFR(Y1068) antibody (Red) and DAPI (blue). Scale bar=20 µm. Shown are representatives from three independent experiments. [file Image_2.tif]

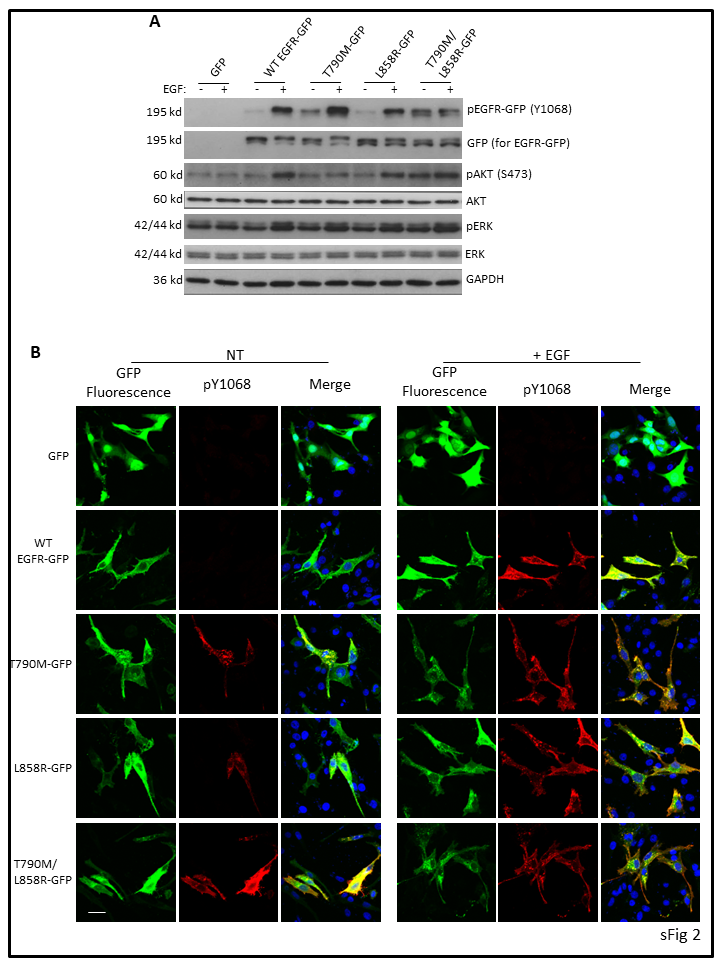

Supplement: Supplementary Figure 3 — EGFR alters the E-cadherin level in WM983B cells. (A) Immunoblottings of indicated proteins from stable WM983B cells expressing GFP or GFP-tagged WT/mutant EGFR. Shown are representative results of three independent experiments. (B) Representative immunofluorescent images of stable WM93B cells immunostained with E-cad antibody (Red) and DAPI (blue). Scale bar=20 µm. Shown are representatives from three independent experiments. [file Image_3.tif]

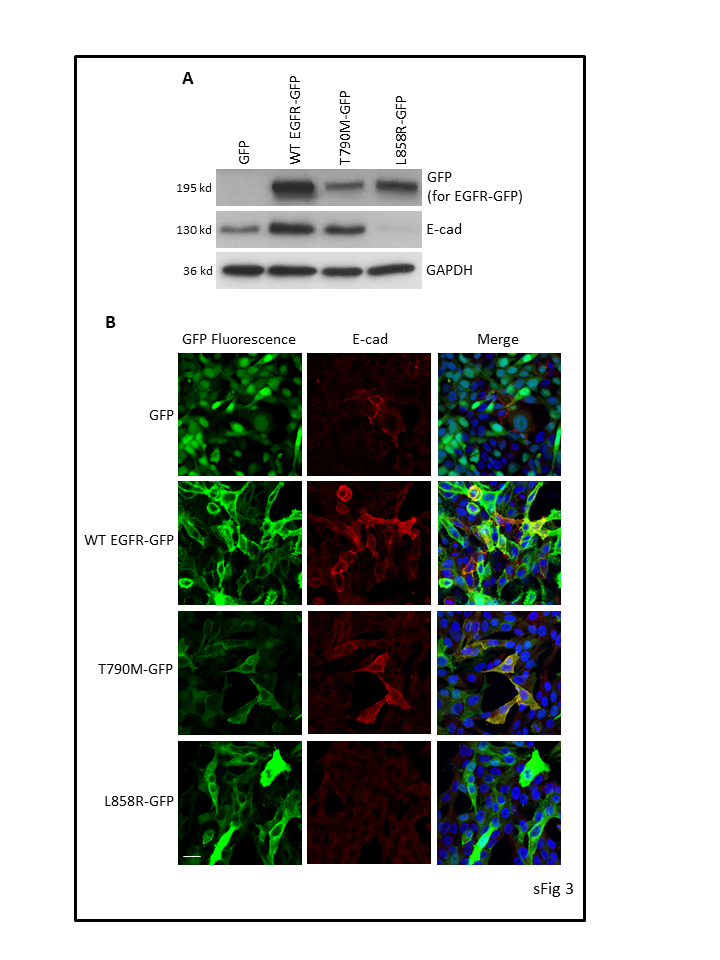

Supplement: Supplementary Figure 4 — Representative images of stable WM983A cells expressing GFP or GFP-tagged EGFR during routine cell cultures. Images were randomly taken under optical microscope at 10x magnification. [file Image_4.tif]

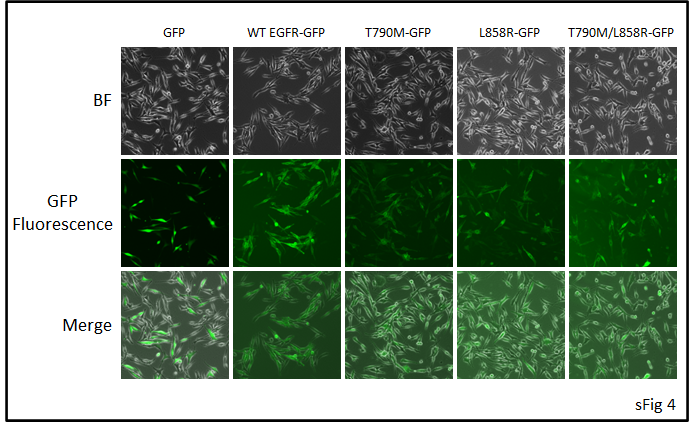

Supplement: Supplementary Figure 5 — Representative images of WM983A and WM983B cells with or without Dox treatment at indicated concentrations for 48h. Images were randomly taken under optical microscope at 10x magnification. [file Image_5.tif]

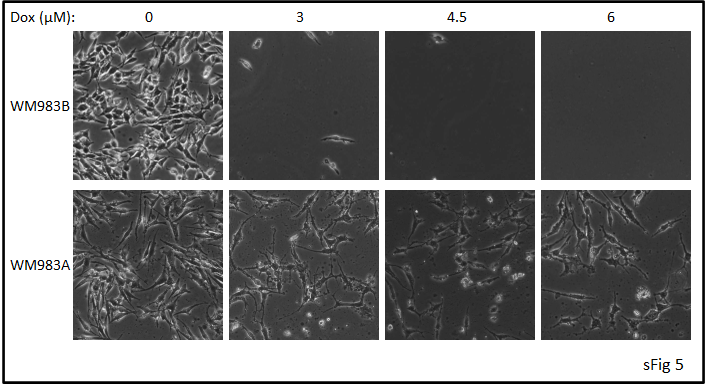

Supplement: Supplementary Figure 6 — EGFR enhances the resistance of WM983B cells to doxorubicin challenge. (A) Representative images of WM983B cells transiently expressing GFP or GFP-tagged WT or mutant EGFR treated with indicated concentrations of Dox for 48h. (B) Immunoblottings of GFP-tagged EGFR proteins from transiently transfected WM983B cells treated with Dox at 3µM or 4µM for 48h. Shown are representatives from three independent experiments. (C) Quantitative results of (B). Data are mean of ± SD of three independent experiments. [file Image_6.tif]

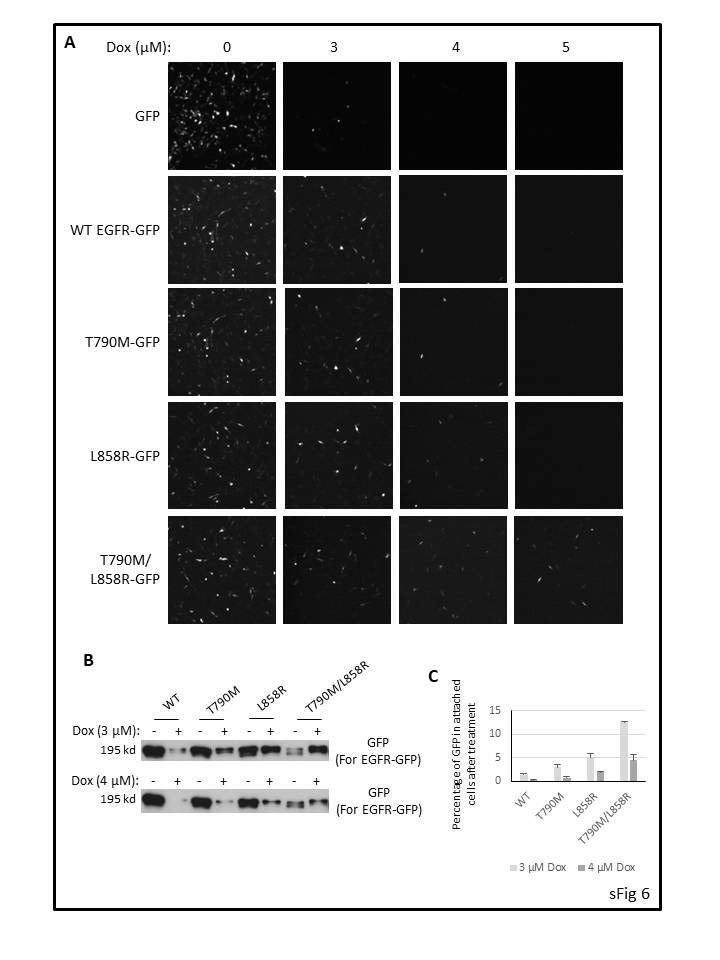

Supplement: Supplementary file 7 [file Image_7.tif]
